# Osseus Lesions in Hemiplegia

**Published:** 1882-03

**Authors:** 


### OSSEOUS LESIONS IN HEMIPLEGIA.

M. Debove has made a few observations, before the Societe Medicale des Hopitaux, on the osseous lesions met with in hemiplegic subjects. As surgeon of the Bicetre Hospital, he had observed several fractures in paralytic subjects, and he noticed, at the same time, that the fracture was always on the side of the hemiplegia. There was reason to believe that the bones on the affected side had undergone some alteration which rendered them more friable. At the autopsy of a hemiplegic who had fractured the humerus, M. Debove was able to observe that not only had the fractured bone undergone this change, but all the bones of that side were affected. In comparing the two sides, he found that the humerus of the paralysed side was lighter than that of the other, the medullary canal was larger, and the substance of the shaft less compact. The Haversian canals were much dilated, and the bone was porous. There was evidence of fatty degeneration. These lesions were sufficient to explain the relative frequence of fractures in hemiplegics. M. Debove observed, however, that these fractures united very rapidly.—*Medical Press and Circular*, November 9, 1881.

